# Function Extension Based Real-Time Wavelet De-Noising Method for Projectile Attitude Measurement

**DOI:** 10.3390/s20010200

**Published:** 2019-12-30

**Authors:** Zhihong Deng, Jinwen Wang, Xinyu Liang, Ning Liu

**Affiliations:** 1 School of Automation, Beijing Institute of Technology, Beijing 100081, China; dzh_deng@bit.edu.cn (Z.D.); 3120185449@bit.edu.cn (X.L.); 2 School of Automation, Beijing Information Science and Technology University, Beijing 100192, China; ning.liu@bistu.edu.cn

**Keywords:** projectile attitude measurement, real-time, wavelet de-noising, function extension

## Abstract

The real-time measurement of the projectile attitude is the key to realize the whole process guidance of the projectile. Due to the high dynamic characteristics of the projectile motion, the attitude measurement is affected by the real-time and accuracy of the gyro signal de-noising. For the nonlinear discontinuity of the conventional extension method in real-time wavelet de-noising, a function extension real-time wavelet de-noising method is proposed in this paper. In this method, a prediction model of gyro signal is established based on the Roggla formula. According to the model, the signal is fitted in the sliding window, and the signal of the same length is predicted to realize the right boundary extension. The simulation and experiment results show that compared with the traditional extension method, the proposed method can in-crease the signal-to-noise ratio (SNR) and the smoothness, and can decrease the attitude mean absolute error (AMAE) and the attitude root mean square error (ARMSE). Moreover, the time delay caused by signal de-noising can be effectively solved. The real-time performance of the attitude measurement can be ensured.

## 1. Introduction

**Figure 1:**
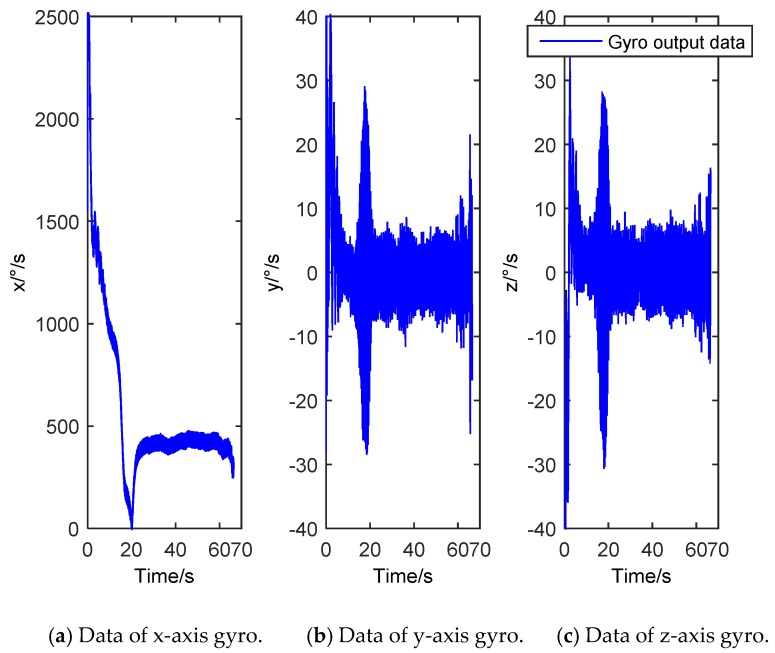
Actual measurement data of gyro.

Gyro is the core component of the projectile attitude measurement. Its accuracy and real-time performance affect the flight and control safety of the projectile. Due to the high dynamic characteristics of projectile in flight, such as high overload and high speed [1], there are many uncertain factors, such as the complex working environment, which cause interference to the measurement of gyro and affect its measurement accuracy [2,3]. Figure 1 shows a group of actual measurement data of gyro. It can be seen that the dynamic characteristics of the projectile flight environment are strong, the data fluctuate greatly, and the introduced nonlinear noise has a great influence on the attitude measurement. Therefore, in the high dynamic environment, it is the key to remove the gyro noise effectively for accurate measurement of the projectile attitude.

For the gyro random noise processing, the main methods at present include: low-pass, high-pass, band-pass or band-stop filter algorithm [4,5], Kalman filter and its derivative algorithm [6]. The wavelet de-noising algorithm overcomes the defects of traditional filter methods, and is with good local characteristics, adaptive characteristics and multi-resolution characteristics [7]. Therefore, wavelet algorithm is widely used in signal de-noising processing.

At present, the wavelet de-noising method mainly focuses on offline batch processing of measurement data [8]. In practical engineering, there is boundary distortion phenomenon in real-time wavelet de-noising of gyro signal [9,10,11], and boundary extension processing is needed for data window. Jianing S proposed the periodic extension technique to eliminate the boundary issue inherent in kernel regression means [12]. Yang A proposed a construction method of wavelet transform matrices with arbitrary decomposition depth while the signal was symmetrically extended [13]. Wei J proposed a novel method for reducing these tile-boundary artifacts. The method employed odd tile sizes (2N + 1 samples) rather than the conventional even tile sizes (2N samples) [14]. Qian et al. proposed neural network prediction model and wavelet packet transform technology into the empirical mode decomposition method to improve the border effect and to enhance the ability of signal de-noising [15]. Common extension methods mainly include: zero extension, periodic extension, symmetric extension and linear extension [8,9,16]. These methods can achieve better results in extension processing of signals with low dynamic performance, but in the high dynamic environment, the output signal of gyro has strong nonlinear characteristics, so the results of these methods are not ideal. Therefore, it is urgent to find a wavelet de-noising boundary extension method for high dynamic application environment.

In this paper, the Roggla formula is introduced to establish the gyro signal prediction model of the projectile attitude measurement. According to this model, a boundary extension method based on function fitting is proposed. The least square method is used to fit the data of the current sliding window and the data of the next sliding window is predicted. A new sliding window with the fitting and prediction results is formed, and the wavelet threshold de-noising is realized. The signal-to-noise ratio (SNR), root mean square error (RMSE), smoothness, attitude mean absolute error (AMAR) and attitude root mean square error (ARMSE) are used as the evaluation indexes of the algorithm. Several common extension methods and the function extension method proposed in this paper are compared and analyzed through simulation and experiment. The results show that the effects of the five evaluation indexes are improved by the function extension method, and the processing results are stable to follow the real signal, and the problem of time delay caused by signal de-noising is solved.

The rest of the paper is organized as follows. In Section 2, we introduce the principle of real-time wavelet threshold de-noising. In Section 3, we establish the prediction model of gyro signal, and propose the function extension method. In Section 4, we analyze the simulation and experiment results. Section 5 draws the conclusions.

## 2. Principle of Real-Time Wavelet Threshold De-Noising

**Figure 2:**
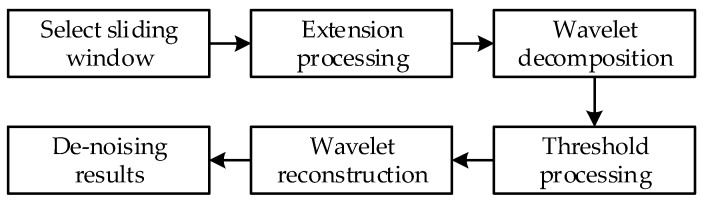
Flowchart of real-time wavelet threshold de-noising.

The real-time wavelet threshold de-noising process is shown in Figure 2 [17].

**Figure 3:**
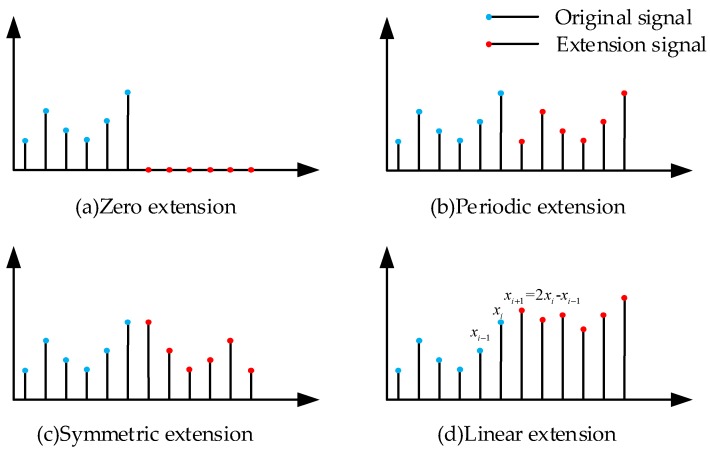
Common boundary extension methods.

In the process of real-time wavelet threshold de-noising, the current signal is located at the right end of the sliding window, and the optimal position of de-noising is located in the middle of the sliding window. Therefore, in this paper only the right boundary extension is performed to shorten the sliding window length and improve the real-time performance of the algorithm. Common boundary extension methods include:

(1) Zero extension. As shown in Figure 3a, this method is easy to produce large step change at the boundary, and introduce large errors.
(2) Periodic extension. As shown in Figure 3b, this method is not applicable when the data changes sharply, which is easy to cause boundary discontinuity.
(3) Symmetric extension. As shown in Figure 3c, this method is not applicable when the data changes violently.
(4) Linear extension. As shown in Figure 3d, this method is not applicable for nonlinear systems.

In the high dynamic environment, the gyro output data changes violently. In order to avoid the problems introduced by the above method, in this paper a boundary extension method is proposed based on function fitting.

## 3. Real-Time Wavelet De-Noising Method Based on Function Extension

### 3.1. Prediction Model of Gyro Signal for Projectile Attitude Measurement

**Figure 4:**
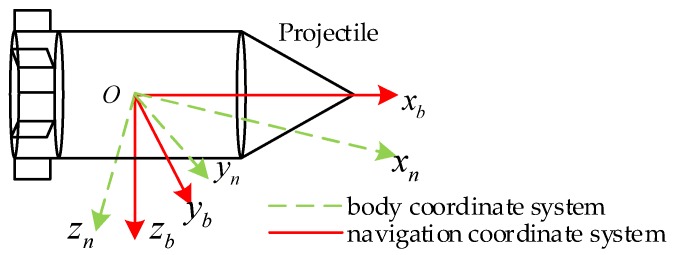
Definition of projectile attitude measurement coordinate system.

The yaw angle ψ, pitch angle θ and roll angle γ of the projectile attitude are a set of Euler angles. The angular position relationship of the body coordinate system ($Ox_{b}y_{b}z_{b}$) relative to the navigation coordinate system ($Ox_{n}y_{n}z_{n}$) are described by the Euler angles. The definition of coordinate system is shown in Figure 4.

Since the latitude and longitude of the projectile from launching point to the landing point do not change much, it can be considered that the gyro output angular rate $\omega_{b}={\left[\begin{array}{ccc} \omega_{bx} & \omega_{by} & \omega_{bz} \end{array}\right]}^{T}$ is equal to the attitude rate $\omega_{nb}$, and the relationship between the Euler angles and $\omega_{b}$ can be obtained as follows:

(1) $$\left[\begin{array}{c} \omega_{bx}\\ \omega_{by}\\ \omega_{bz} \end{array}\right]=\left[\begin{array}{ccc} -\sin\left(\theta \right) & 0 & 1\\ \cos\left(\theta \right)\sin\left(\gamma \right) & \cos\left(\gamma \right) & 0\\ -\cos\left(\theta \right)\cos\left(\gamma \right) & \sin\left(\gamma \right) & 0 \end{array}\right]\left[\begin{array}{c} \dot{\psi }\\ \dot{\theta }\\ \dot{\gamma } \end{array}\right]$$

Thus:

(2) $$\left\{ \begin{array}{l} \omega_{bx}=-\sin\left(\theta \right)\dot{\psi }+\dot{\gamma }\\ \omega_{by}=\cos\left(\theta \right)\sin\left(\gamma \right)\dot{\psi }+\cos\left(\gamma \right)\dot{\theta }\\ \omega_{bz}=-\cos\left(\theta \right)\cos\left(\gamma \right)\dot{\psi }+\sin\left(\gamma \right)\dot{\theta } \end{array}\right.$$

Due to the high dynamic characteristics of the projectile, the distance spread is much larger than the direction spread, and the change of yaw angle is very little, the change is generally only about 2° [18]. Therefore, it can be assumed that $\dot{\psi }=0$. Thus the prediction model of gyro signal for projectile attitude measurement can be obtained by the simplified formula (2):

(3) $$\left\{ \begin{array}{l} \omega_{bx}=\dot{\gamma }\\ \omega_{by}=\cos\left(\gamma \right)\dot{\theta }\\ \omega_{bz}=\sin\left(\gamma \right)\dot{\theta } \end{array}\right.$$

After the projectile is ejected, the angular rate of the projectile self-rotation is exponentially attenuated, and the process can be described by the Roggla formula [19]:

(4) $$\dot{\gamma }\left(t\right)=\omega_{g}e^{\left(-0.075k\frac{L_{d}D^{3}}{A}t\right)}$$

where, $\dot{\gamma }\left(t\right)$ is the angular rate of the projectile self-rotation, $\omega_{g}$ is the angular rate of initial self-rotation, $L_{d}$ is the length of the projectile, *D* is the diameter of the projectile, *A* is the polar moment of inertia of the projectile, and *k* is the correction coefficient.

### 3.2. Function Extension Method

It can be known from formulas (3) and (4) that the angular motion of the gyro-sensitive projectile has a certain regularity. Therefore, the boundary extension method can be designed according to this regularity.

Set: $a=\omega_{g}$, $b=0.075k\frac{L_{d}D^{3}}{A}$, then formula (4) can be abbreviated as:

(5) $$\dot{\gamma }\left(t\right)=ae^{-bt}$$

By integral of formula (5), the following formula can be obtained:

(6) $$\gamma \left(t\right)=\int \dot{\gamma }\left(t\right)dt=\int ae^{-bt}dt=-\frac{a}{b}e^{-bt}+c$$

**Figure 5:**
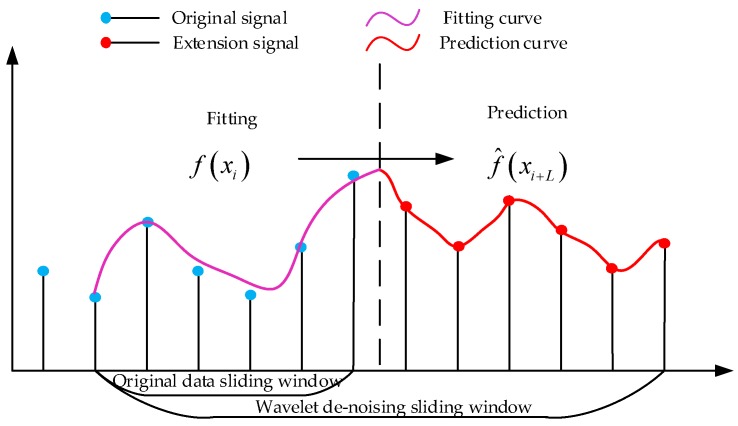
Function extension method.

Combining formulas (3), (5) and (6), a function extension method can be designed and its basic idea is shown in Figure 5.

It can be seen from Figure 5 that the data in the sliding window is fitted by the method. The prediction data of the right boundary extension can be obtained.

In the process of function extension, the sliding window length is *L*. In the sliding window, the change of pitch angle is small, and it can be considered that $d=\dot{\theta }$ is constant. Therefore, simplified formula (3) can obtain as follows:

(7) $$\left\{ \begin{array}{l} \omega_{bx}=ae^{-bt}\\ \omega_{by}=d\cos\left(-\frac{a}{b}e^{-bt}+c\right)=d\cos\left(-\frac{\omega_{bx}}{b}+c\right)\\ \omega_{bz}=d\sin\left(-\frac{a}{b}e^{-bt}+c\right)=d\sin\left(-\frac{\omega_{bx}}{b}+c\right) \end{array}\right.$$

According to formula (7), the function is fitted and the right boundary data is predicted. The specific operations are as follows:

For the *x*-axis, the logarithm on both sides as follows:

(8) $$\ln\left(\omega_{bx}\right)=\ln\left(a\right)-bt$$

For the sliding window with length *L*, the least square method is used for curve fitting. $W_{x}={\left[\begin{array}{ccc} \ln\left(\omega_{bx,1}\right) & \ldots & \ln\left(\omega_{bx,L}\right) \end{array}\right]}^{T}$, $T_{x}=\left[\begin{array}{cc} 1 & -t_{1}\\ \ldots & \ldots \\ 1 & -t_{L} \end{array}\right]$, $x={\left[\begin{array}{cc} \ln\left(a\right) & b \end{array}\right]}^{T}$. According to the least square method:

(9) $$x={\left(T_{x}^{T}T_{x}\right)}^{-1}T_{x}^{T}W_{x}$$

For the original data sliding window with length *L*, the extension length is also *L*, then a new sliding window data are formed by *x*-axis prediction and fitting results as follows:

(10) $${\left[\begin{array}{cccc} {\hat{\omega }}_{bx,1} & \ldots & {\hat{\omega }}_{bx,L} & \begin{array}{cc} \begin{array}{cc} {\hat{\omega }}_{bx,L+1} & \ldots \end{array} & {\hat{\omega }}_{bx,2L} \end{array} \end{array}\right]}^{T}={\left[\begin{array}{cccc} ae^{-bt_{1}} & \ldots & ae^{-bt_{L}} & \begin{array}{cc} \begin{array}{cc} ae^{-bt_{L+1}} & \ldots \end{array} & ae^{-bt_{2L}} \end{array} \end{array}\right]}^{T}$$

For the *y*-axis, the parameter *b* can be obtained by fitting the *x*-axis data. Set $\omega_{x}=-\frac{\omega_{bx}}{b}$, then $\omega_{by}=d\cos\left(\omega_{x}+c\right)$. According to the formula of trigonometric function, the formula (7) can be expanded as follows:

(11) $$\omega_{by}=d\cos\left(\omega_{x}+c\right)=d\cos\left(c\right)\cos\left(\omega_{x}\right)-d\sin\left(c\right)\sin\left(\omega_{x}\right)$$

The least square method is also used for curve fitting, $W_{y}={\left[\begin{array}{ccc} \omega_{by,1} & \ldots & \omega_{by,L} \end{array}\right]}^{T}$, $T_{y}=\left[\begin{array}{cc} \cos\left(\omega_{x,1}\right) & -\sin\left(\omega_{x,1}\right)\\ \ldots & \ldots \\ \cos\left(\omega_{x,L}\right) & -\sin\left(\omega_{x,L}\right) \end{array}\right]$, $y={\left[\begin{array}{cc} d\cos\left(c\right) & d\sin\left(c\right) \end{array}\right]}^{T}$. According to the least square method:

(12) $$y={\left(T_{y}^{T}T_{y}\right)}^{-1}T_{y}^{T}W_{y}$$

For the original data sliding window with length *L*, the extension length is also *L*, then a new sliding window data are formed by *y*-axis prediction and fitting results as follows:

(13) $$\begin{array}{l} {\left[\begin{array}{cccc} {\hat{\omega }}_{by,1} & \ldots & {\hat{\omega }}_{by,L} & \begin{array}{cc} \begin{array}{cc} {\hat{\omega }}_{by,L+1} & \ldots \end{array} & {\hat{\omega }}_{by,2L} \end{array} \end{array}\right]}^{T}=[\begin{array}{ccc} d\cos\left(-\frac{ae^{-bt_{1}}}{b}+c\right) & \ldots & d\cos\left(-\frac{ae^{-bt_{L}}}{b}+c\right) \end{array}\\ {\begin{array}{ccc} d\cos\left(-\frac{ae^{-bt_{L+1}}}{b}+c\right) & \ldots & d\cos\left(-\frac{ae^{-bt_{2L}}}{b}+c\right) \end{array}]}^{T} \end{array}$$

For the *z*-axis, the *y*-axis curve can be used directly to fit the parameters. For *z*-axis, *y*-axis curve fitting parameters can be used directly.

**Figure 6:**
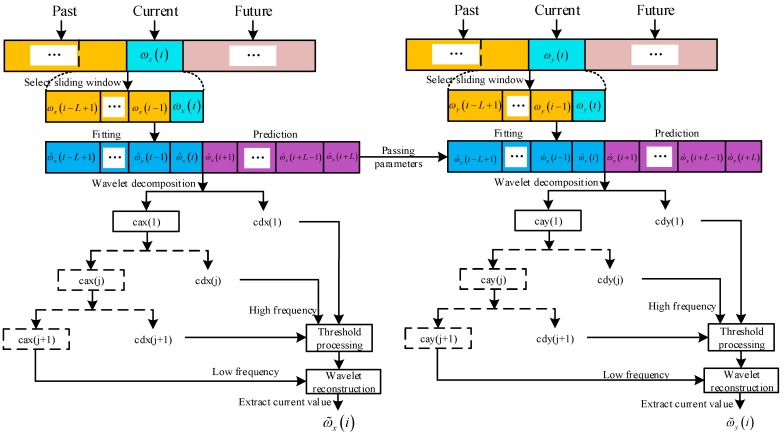
Flowchart of real-time wavelet threshold de-noising based on function extension.

The flowchart of real-time wavelet threshold de-noising based on function extension method is shown in Figure 6.

In Figure 6, ca represents low frequency wavelet coefficient, cd represents high frequency wavelet coefficient, and threshold processing mainly deals with high frequency coefficients, and j is the number of decomposition layers. The *z*-axis de-noising process is the same as the *y*-axis.

After the boundary extension, the wavelet decomposition, threshold processing, wavelet reconstruction and de-noising results are extracted by combining Figure 2 and Figure 6.

## 4. Algorithm Verification and Result Analysis

### 4.1. Evaluation Indexes of Algorithm

For the simulation, the SNR and root mean square error (RMSE) are used as evaluation indexes because of the true value [20].

(1) SNR:

(14) $$SNR=10log_{10}\left(\frac{\sum_{N}\omega^{2}\left(t\right)}{\sum_{N}{\left(\omega \left(t\right)-\tilde{\omega }\left(t\right)\right)}^{2}}\right)$$

where, ω(t) represents the true value of the signal, $\tilde{\omega }\left(t\right)$ represents the filtering result, *N* represents the signal length, and the *SNR* unit is decibel (db). The larger the *SNR* is, the more useful signals are, and the better the de-noising effect is.

(2) RMSE:

(15) $$RMSE=\sqrt{\frac{1}{N}\sum_{N}{\left(\omega \left(t\right)-\tilde{\omega }\left(t\right)\right)}^{2}}$$

The smaller the RMSE is, the closer the filtering result is to the real signal, and the better the de-noising effect is.

For the experiment, it is difficult to obtain the true value. The smoothness is used as the evaluation index of the de-noising effect, and the smoothness can reflect the smoothness of the de-noising signal. The smaller the smoothness, the smoother the curve and the better the de-noising effect. Its expression is as follows:

(16) $$r=\frac{\sqrt{\sum_{N-1}{\left(\tilde{\omega }\left(t+1\right)-\tilde{\omega }\left(t\right)\right)}^{2}}}{\sqrt{\sum_{N-1}{\left(\bar{\omega }\left(t+1\right)-\bar{\omega }\left(t\right)\right)}^{2}}}$$

where, $\bar{\omega }\left(t\right)$ represents the original signal.

When de-noising based on the function extension wavelet is completed, the single-sample algorithm is used to the attitude calculation, and the AMAE and the ARMSE are defined as the evaluation indexes.

(1) AMAE:

(17) $$AMAE=\frac{1}{3N}\left(\sum_{N}\left|\theta \left(t\right)-\theta_{L}\left(t\right)\right|+\sum_{N}\left|\gamma \left(t\right)-\gamma_{L}\left(t\right)\right|+\sum_{N}\left|\psi \left(t\right)-\psi_{L}\left(t\right)\right|\right)$$

where, $\left[\begin{array}{ccc} \theta \left(t\right) & \gamma \left(t\right) & \psi \left(t\right) \end{array}\right]$ is the result of attitude calculation using the true value of the signal; $\left[\begin{array}{ccc} \theta_{L}\left(t\right) & \gamma_{L}\left(t\right) & \psi_{L}\left(t\right) \end{array}\right]$ is the result of attitude calculation after de-noising by window length *L* function extension.

(2) ARMSE:

(18) $$ARMSE=\frac{1}{3\sqrt{N}}\left(\sqrt{\sum_{N}{\left(\theta \left(t\right)-\theta_{L}\left(t\right)\right)}^{2}}+\sqrt{\sum_{N}{\left(\gamma \left(t\right)-\gamma_{L}\left(t\right)\right)}^{2}}+\sqrt{\sum_{N}{\left(\psi \left(t\right)-\psi_{L}\left(t\right)\right)}^{2}}\right)$$

### 4.2. Simulation Comparison of Different Extension Methods

Simulation conditions: under standard meteorological conditions, the full ballistic attitude data is simulated. The initial rotation rate of the projectile is 10 r/s, the sampling frequency is 1000 Hz, and the gyro bias stability is 10°/h. According to multiple simulations, the relevant parameters are selected as shown in Table 1.

**Figure 7:**
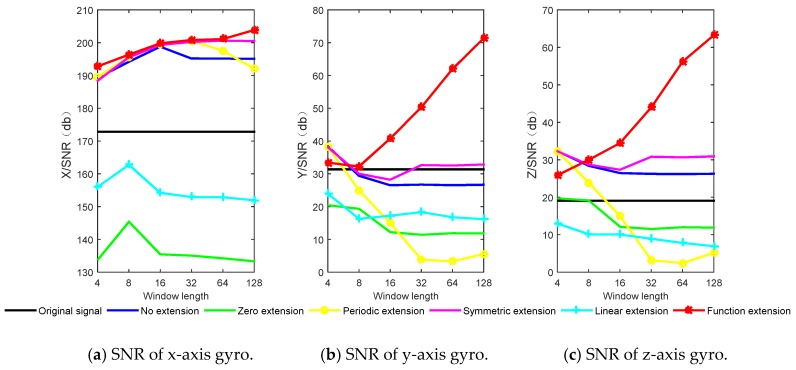
Simulation results of SNR for different extension methods.

**Figure 8:**
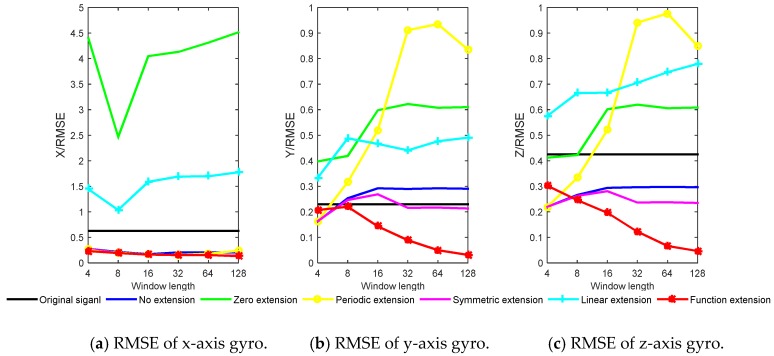
Simulation results of RMSE for different extension methods.

According to the characteristics of the wavelet transform, the length of sliding window *L* = 4, 8, 16, 32, 64, 128 are selected. The real-time wavelet threshold de-noising is performed on the three axial gyro data by using the methods of no extension, zero extension, periodic extension, symmetric extension, linear extension and function extension respectively. The simulation results of the six extension methods and the original signal SNR and RMSE are shown in Figure 7 and Figure 8.

It can be seen from Figure 7 and Figure 8 that the function extension method is superior to other methods, and with the increase of window length, the SNR increases, and RMSE decreases. For the three axial gyros, the results of zero extension and linear extension are not as good as the original signal, which indicates that the two methods are not applicable in high dynamic environment. For the *y*-axis and the *z*-axis, the effect of no extension and periodic extension is poor, which indicates that the real-time wavelet threshold de-noising for these two axes needs to be extended, and the extension method has a great influence on the de-noising effect. The performance improvement (PI) is defined as the increase percentage of SNR for different extension methods relative to the original signal. The window length *L* = 128 is taken as an ex-ample. The SNR and PI results of different methods are shown in Table 2.

It can be seen from Table 2 that compared with the original signal, for the *x*-axis, the zero extension and the linear extension do not improve the SNR, and the performance of the function extension method increases the most. For the *y*-axis, only symmetric extension and function extension have PI. Due to the high dynamic environment, the *y*-axis data changes drastically in a short time, other extension methods are not suitable, and the PI of the function extension method is 127.69%, which proves the practicability of this method. For the *z*-axis, no extension, symmetric extension and function extension have PI.

**Figure 9:**
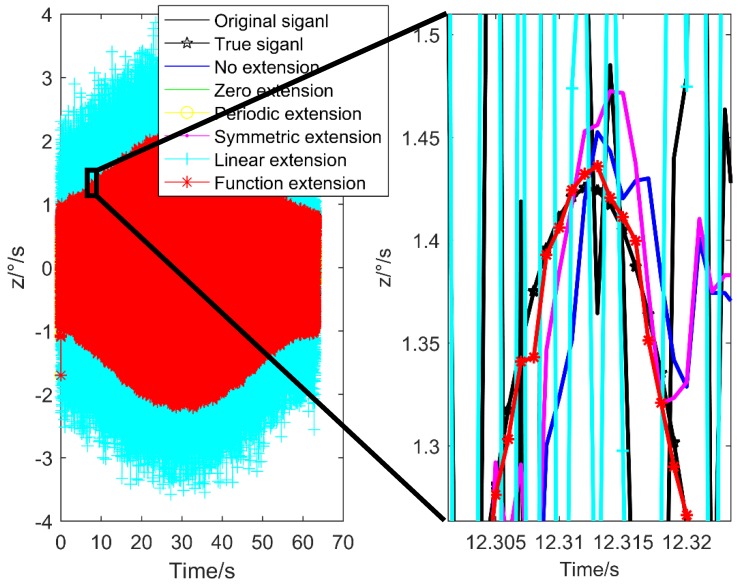
De-noising results of different extension methods.

In order to further illustrate the applicability of the pro-posed method, taking the *z*-axis signal as an example, the de-noising results using different extension methods are shown in Figure 9.

It can be seen from Figure 9 that for the *z*-axis data change drastically, there will be signal delay phenomenon by using other extension methods, and the de-noising result cannot follow the original signal change normally. Because the high-frequency wavelet coefficients are set to zero by the wavelet threshold. The high-frequency component of the signal has a large attenuation amplitude, the time delay is gradually increased, and the signal amplitude is gradually decreased, and the dynamic response performance is obviously reduced. The function extension method can follow the original signal normally. Because the function fitting result is used to the wavelet threshold de-noising. In the process of extension processing, some high-frequency signals have been removed, which ensures the accuracy of wavelet threshold de-noising.

### 4.3. Experiment Comparison of Different Extension Methods

**Figure 10:**
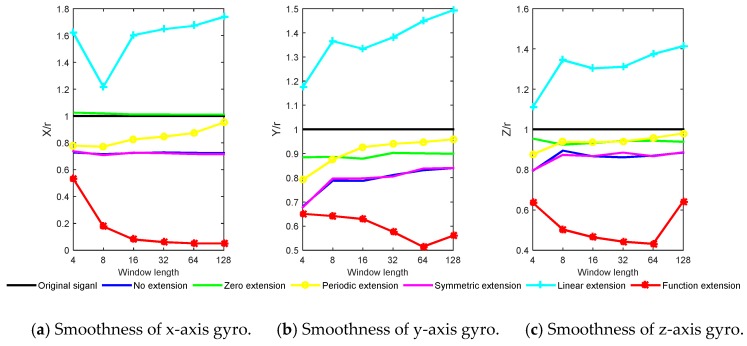
Experiment results of smoothness for different extension methods.

According to the actual experiment data of projectile, the performance of different methods are verified, and the parameter setting is the same as the simulation part. Smoothness is used as an evaluation index for de-noising effects. The real-time wavelet threshold de-noising is performed on the three axial gyro data by using the six extension methods respectively. The simulation results of the six extension methods and the original signal smoothness are shown in Figure 10.

It can be seen from Figure 10 that the function extension method is superior to other methods, and with the window length increases, the smoothness decreases within 64. Because the actual projectile motion is more complicated than the simulation motion, it may contain regular precession, general angular velocity motion and random angular vibration. Therefore, the simulation results are different from the experiment results, but in terms of the overall trend, the simulation results and experiment results have certain commonality. For the three axial gyro de-noising results, the linear extension results are not as good as the original signal, and the periodic extension and zero extension are not good, which is similar to the simulation results.

**Figure 11:**
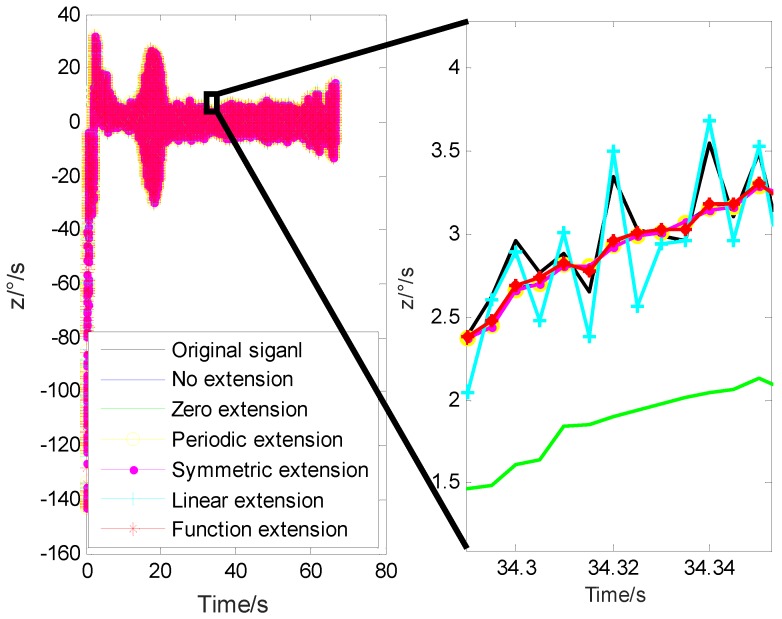
De-noising results of different extension methods for the actual data.

In order to further illustrate the applicability of the proposed method, taking the *z*-axis signal as an example, the de-noising results using different extension methods are shown in Figure 11.

It can be seen from Figure 11 that although there are some differences between the experiment and the simulation, the effect of wavelet threshold de-noising using different extension methods is similar.

### 4.4. Real-Time Verification Comparison of Different Extension Methods

The AMD A8-4599M processor with the main frequency of 1.90 GHz is used for simulation in MATLAB R2014b. The actual experiment data is used as the simulation object, and the single calculation period is used as the evaluation index. The specific results are shown in Table 3.

It can be seen from Table 3 that with the window length increases, the single calculation period of the different extension methods does not increase significantly.

The results of simulation and experiment show that the function extension method is superior to other extension methods.

### 4.5. Accuracy of Attitude Calculation Comparison of Different Window Length

**Figure 12:**
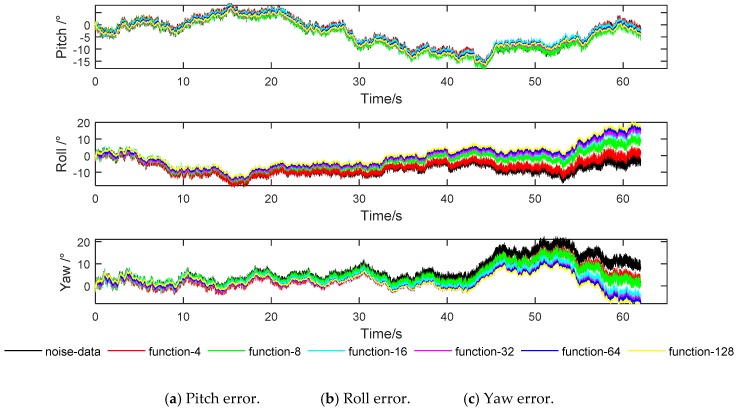
Attitude calculation error of different window length function extension.

In order to facilitate the description, the function extension of six kinds of length windows *L* = 4, 8, 16, 32, 64, 128 are defined as “function-4”, “function-8”, “function-16”, “function-32”, “function-64”, “function-128”; the original noise signal is de-fined as “noise-data”, and the simulation results are shown in Figure 12.

It can be seen from Figure 12 that the noise has a great influence on the attitude calculation result. De-noising by function extension method can weaken the influence of noise, and the attitude calculation results are different with different window length selection. According to Figure 12, the AMAE and ARMSE are shown in Table 4.

It can be seen from Table 4 that AMAE and ARMSE of the original signal attitude calculation are relatively large, indicating that the noise has a great influence on the attitude calculation, and it is necessary to de-noise the gyro signal. Compared with the original signal, AMAE and ARMSE are significantly reduced by wavelet de-noising based on function extension, which indicates that the function extension method has practicality. The window length affects the function extension processing effect, and when *L* = 16, AMAE and ARMSE are the smallest and the de-noising effect is the best.

## 5. Conclusions

In this paper, the problem of poor wavelet threshold de-noising effect of gyro signal is studied by the conventional extension method in high dynamic environment. According to the flight characteristics of the projectile, the gyro signal prediction model of the projectile attitude measurement is established by introducing the Roggla formula. A boundary extension method of function fitting is proposed. The least square method is used to fit the data of the sliding window, and the prediction of the next sliding window data is realized. The fitting and prediction results form a new sliding window, which is de-noised by wavelet threshold. Simulation and experiment results show that:

(1) Compared with other extension methods, the gyro signal output characteristics of the projectile attitude measurement is combined, the SNR is improved greatly, the RMSE of the signal is reduced, the smoothness of the signal increased, and the AMAE and ARMSE are reduced, and the real-time performance of the algorithm can be guaranteed by the function extension method, which has great research significance in practical applications.
(2) For the gyro *y*-axis and *z*-axis data, other extension methods will produce time delay, while the function extension method can stably follow the signal, effectively removing random noise and ensuring the accuracy of gyro data, which have a strong practical application value.

## Figures and Tables

**Table 1:** Simulation parameters.

	Parameters	Angle Random Walk	Wavelet Base	Decomposition Layers	Threshold Principle	Threshold Function
Axial						
*x*		$1.5{}^{\circ}/\sqrt{h}$	Bior 2.6	5	Fixed threshold	Soft threshold
*y*		$1{}^{\circ}/\sqrt{h}$	Coif 5	4	Unbiased Risk Estimate	
*z*		$2{}^{\circ}/\sqrt{h}$				

**Table 2:** SNR and PI results of different extension methods.

Methods	Axial					
	*x*		*y*		*z*	
	SNR/db	PI	SNR/db	PI	SNR/db	PI
Original signal	172.8116	0%	31.3578	0%	19.0873	0%
No extension	195.0846	12.89%	26.6953	0%	26.2669	37.61%
Zero extension	133.3205	0%	11.8373	0%	11.9050	0%
Periodic extension	192.0808	11.15%	5.5727	0%	5.2239	0%
Symmetric extension	200.4983	16.02%	32.8780	4.85%	30.9326	62.06%
Linear extension	151.9784	0%	16.2021	0%	6.9540	0%
Function extension	**203.8435**	**17.96%**	**71.3984**	**127.69%**	**63.2918**	**231.59%**

**Table 3:** The single calculation period results of different extension methods (ms).

Methods	Window Length					
	4	8	16	32	64	128
No extension	4.783	4.501	4.235	4.059	4.102	4.414
Zero extension	4.335	4.266	4.259	4.262	4.487	4.876
Periodic extension	4.979	4.189	4.328	4.682	4.598	4.341
Symmetric extension	4.358	4.133	4.162	4.608	4.452	4.629
Linear extension	4.860	5.479	5.377	5.414	5.725	5.767
Function extension	**4.098**	**4.991**	**4.637**	**5.923**	**5.674**	**6.108**

**Table 4:** AMAE and ARMSE results of different window length function extension (°).

	Length	Noise-Data	Function-4	Function-8	Function-16	Function-32	Function-64	Function-128
Indexes								
AMAE		6.7038	5.5681	5.1957	**4.6410**	4.6811	4.6848	4.8271
ARMSE		8.0766	6.9299	6.5271	**5.9079**	5.9379	5.9163	6.0618
